# Self-harm and rurality in Canada: an analysis of hospitalization data from 2015 to 2019

**DOI:** 10.1007/s00127-023-02463-7

**Published:** 2023-04-08

**Authors:** Newsha Mahinpey, Nathaniel J. Pollock, Li Liu, Gisèle Contreras, Wendy Thompson

**Affiliations:** 1 Centre for Surveillance and Applied Research, Public Health Agency of Canada, Ottawa, ON Canada; 2 Faculty of Medicine and Dentistry, University of Alberta, Edmonton, Canada; 3 School of Arctic and Subarctic Studies, Labrador Campus, Memorial University, Happy Valley-Goose Bay, NL Canada

**Keywords:** Suicide, Self-inflicted injury, Rural, Urban, Surveillance, Administrative data, Health disparities

## Abstract

**Purpose:**

The incidence of self-harm is an important indicator in suicide surveillance and a target outcome for suicide prevention. Self-harm rates vary by geographic location and rurality appears to be a risk factor. The objectives of this study were to estimate rates of self-harm hospitalization in Canada over a 5-year period by sex and age group, and examine relationships between self-harm and rurality.

**Methods:**

Hospitalizations related to self-harm were identified in a national dataset (the Discharge Abstract Database) for all patients aged 10 years or older who were discharged from hospital between 2015 and 2019. Self-harm hospitalization rates were calculated and stratified by year, sex, age group, and level of rurality, as measured using the Index of Remoteness. A Poisson regression was fit to estimate rate ratios for the levels of rurality.

**Results:**

Rates of self-harm hospitalization were higher for females than males across all levels of rurality and increased with each level for both sexes, except for among young males. The widest rural-to-urban disparities were observed for the 10–19 and 20–34-year old age groups. Females aged 10–19 in very remote areas had the highest self-harm hospitalization rate.

**Conclusion:**

The rate of self-harm hospitalization in Canada varied by sex, age group, and level of rurality. Clinical and community-based interventions for self-harm, such as safety planning and increased access to mental health services, should be tailored to the differential risks across geographic contexts.

**Supplementary Information:**

The online version contains supplementary material available at 10.1007/s00127-023-02463-7.

## Introduction

Self-harm has a complex etiology that includes biological, social, psychological, and environmental dimensions [1]. It is defined as acts of "intentional self-poisoning or injury, irrespective of the apparent purpose” [2]. Suicide attempts and non-suicidal self-injuries are both forms of self-harm, and are risk factors for hospitalization and suicide death [3]. Self-harm is a target outcome in suicide prevention [4].

In Canada, the rate of self-harm hospitalization is one the main indicators used in suicide and mental health surveillance [5], and is a proxy for suicide attempts [6]. Between 1994 and 2014, the self-harm hospitalization rate fell from 87 to 50 hospitalizations per 100,000 population [7]. In 2018, the rate was about two times higher among females than males (60.1 vs. 34.4 per 100,000) [6]. This pattern contrasts with suicide mortality, which has rates that are about three times higher among males [8], and is a paradox evident in many high-income countries [9]. In 2018, self-harm was one of the ten leading causes of hospitalization in Canada among people aged 15–19 and 20–24 years [6]. Identifying populations at elevated risk of self-harm is a key goal in suicide prevention strategies [4].

Rurality is an important contextual factor in the epidemiology of self-harm [10]. In Canada, rural–urban differences have been observed for many non-communicable diseases, injuries, and causes of death. [11]. Much of the research on suicide-related outcomes and rurality in Canada has focused on suicide mortality [12–15], specific age groups [14, 16–19], or provinces/territories [15–18, 20], with limited evidence on rural–urban differences in hospitalization [16, 17, 21].

A recent study found that suicide was the second leading cause of preventable death in remote communities [22]. Other Canadian studies have reported conflicting results [12–17, 19–21, 23]: some found that rates of suicide [13, 15, 20, 21] and self-harm [16, 17, 19, 21] were higher in rural regions, while others reported no rural–urban differences [12, 14, 18].

The rural–urban pattern in Canada is different than in the United Kingdom and Ireland, where rates of self-harm and suicide tend to be higher in urban areas after adjusting for socio-economic deprivation [24]. In Canada, there is a pronounced gradient in rates of self-harm and suicide across levels of deprivation [25, 26], though how this influences rural–urban differences is less clear. A national prospective cohort study of adults aged 25 or older found that after controlling for individual and area-level factors including income and deprivation, rates of suicide among males were higher in rural and small urban areas than in the largest cities; results for females varied [12]. These findings were corroborated by a recent study from one Canadian province (Ontario), which reported an increased risk of suicide among males, but not females, in rural areas. However, rural males and females had higher risks for self-harm hospitalization than urban populations, even after deprivation and other social factors were taken into account [21].

One of the challenges for epidemiological studies in rural health is that rurality is not measured in a consistent or standardized way; this affects the comparability of results, especially across settings [24, 27]. Some definitions of rurality use binary rural/urban categories based on population density [28]. Another common approach is to stratify populations across “metropolitan influenced zones,” allowing differentiation between areas outside of census metropolitan areas (urban areas with a population of at least 100,000) and census agglomerations (areas with population of at least 10,000) [29, 30]. A limitation of both definitions is that they do not distinguish between rural and remote regions. This is particularly important in Canada, where many small and low density communities are distributed over large geographical areas, notably in the Arctic and subarctic or northern regions [31].

The objectives of this study were to: (1) estimate rates of hospitalization due to self-harm at a subnational level over a five-year period (2015–2019) by sex and age group; and (2) assess the impact of rurality on self-harm hospitalization rates, by sex and age group. In the context of suicide prevention, evidence about the relationship between self-harm and rurality can help inform public health interventions and social policies.

## Methods

### Data source and primary outcome

The Canadian Institute for Health Information’s Discharge Abstract Database (DAD) was the main source of data in this study. The DAD is comprised of demographic, administrative, and clinical information about patients discharged from acute care. Hospital-based care is part of the universal, publicly funded healthcare system in Canada. Because acute care facilities in all provinces and territories except Quebec are required to submit data to the DAD, it is a comprehensive and robust dataset of hospital discharges. The DAD does not include patients who visited the emergency department but were not admitted to hospital. That is, a patient with an episode of self-harm who presented to the emergency department, received an assessment and treatment, and was then discharged, would not be included in the DAD. Rather, the DAD captures the less frequent, but typically more medically serious self-harm events that may require interventions such as trauma management or involuntary admission for safety or for psychiatric care.

For the present study, we used a subnational version of the DAD that covered 77% of the 2016 population of Canada. Data from two northern territories, Northwest Territories and Yukon, were excluded because population estimates by sex and age group at the census subdivision level (CSD; municipalities or small areas equivalent to a municipality) [32], which were needed for stratified analysis by level of rurality, were not available.

The DAD is coded with the *International Statistical Classification of Diseases and Related Health Problems, 10th Revision, Canada* (ICD-10-CA) [33]. The primary outcome in this study was self-harm hospitalizations, identified by ICD-10 diagnosis codes for intentional self-injuries (X60-X84 and Y87.0). These codes include suicide attempts and non-suicidal self-injuries because it is not possible to distinguish suicidal from non-suicidal forms of self-harm in the discharge diagnosis fields. Patients who died in hospital because of their injuries (*n* = 897) were excluded from all analyses; cases from jurisdictions that provided data but lacked record-level CSD information (*n* = 2137) were excluded from the rurality analysis.

We analyzed data on all patients aged 10 or older who were discharged from a hospital with a diagnosis of self-harm between April 1, 2015 and March 31, 2020. DAD uses fiscal years (April 1 to March 31), rather than calendar years. The fivefiscal-year study period was chosen to establish pre-COVID-19 baseline hospitalization rates.

### Rurality

Rurality was measured using the *Index of Remoteness* [34] developed by Statistics Canada. Unlike other measures of rurality [35], this index captures geographic remoteness by considering population size and distance from points of service provision for each census subdivision [36]. By using population size and accessibility to other areas, the Index of Remoteness attempts to differentiate between urban, rural, and remote regions. The Index is based on 2016 Census data and geographic boundaries.

Each census subdivision is assigned a remoteness index (RI) value ranging from 0 to 1, with 0 being the least remote (and most urban) and 1, the most remote. The most remote areas are those with the smallest populations and low accessibility to population centers. A previous study categorized RI scores according to five natural breaks, while also considering the number of census subdivisions and the population distribution in each category [22, 37]. The categories are: easily accessible (RI score < 0.1500), accessible (0.1500 to 0.2888), less accessible (0.2889 to 0.3898), remote (0.3899 to 0.5532), and very remote (> 0.5532). The Index of Remoteness is mapped to DAD data via the census subdivision of each discharged patient’s place of residence.

### Analyses

We calculated crude and age-standardized self-harm hospitalization rates overall, and by year, sex, age group, and rurality. We used a binary variable for sex (male/female) because the number of hospitalizations with sex coded as non-binary (including transgender, intersex, sex unknown, or other) accounted for less than 0.1% of all cases; these cases could not be disaggregated and so were excluded from the analyses. We used 5-year age groups for the overall and sex-stratified analysis. To avoid small cell counts for the rurality analysis, we used four life course age groups (10–19, 20–34, 35–64, 65 +). Population data were drawn from Statistics Canada’s annual demographic estimates for the denominators in rate calculations; the 2011 Canadian Census population was used as the standard population. A Poisson regression was fit to estimate rate differences and rate ratios with 95% confidence intervals for the effect of rurality on self-harm hospitalization, using the least remote area (easily accessible) as the reference group. Analyses were conducted in SAS Enterprise Guide version 7.1 (SAS Institute, Cary, NC, USA).

## Results

### Self-harm hospitalization rates by sex and age group

We identified 68,378 self-harm hospitalizations between 2015 and 2019 (Table 1). For the five-year period, the overall age-standardized self-harm hospitalization rate was 54.4 hospitalizations per 100,000. The annual rate fell slightly from 55.4 per 100,000 in 2015 to 52.0 per 100,000 in 2019, though confidence intervals between consecutive years often overlapped, which suggested that rates were not always significantly different. The five-year age-standardized rate for females was 70.2 per 100,000 (95% CI 69.5–70.8); the rate for males was 39.9 per 100,000 (95% CI 39.4–40.4). Each year during the period, rates were higher among females than males (Table 2).

**Table 1:** Count and percentage distribution of self-harm hospitalizations and population, by sex, age group and rurality, Canada (excluding Quebec, Northwest Territories, and Yukon) 2015–2019

Variable	Population^a^	Self-harm hospitalizations	
		Count	%
Overall	25,151,783	68,378	100.0
Sex			
Female	12,728,821	43,766	64.0
Male	12,422,963	24,612	36.0
Age group			
10–19	3,201,694	18,345	26.8
20–34	5,861,030	20,139	29.5
35–64	11,480,207	25,069	36.7
65+	4,608,853	4825	7.1
Level of rurality^b^			
Easily accessible area	16,285,585	32,132	48.5
Accessible area	5,537,561	18,211	27.5
Less accessible area	2,127,191	8332	12.6
Remote area	1,018,914	5235	7.9
Very remote area	182,531	2331	3.5

^a^Population estimated based on 5-year average; ^b^The total count of self harm hospitalizations used for analysis of rural (denominator in %) was 66,241

**Table 2:** Self-harm hospitalization rate per 100,000 population, Canada (excluding Quebec, Northwest Territories, and Yukon), 2015–2019

	Fiscal year	Count	Crude rate per 100,000 (95% CI)	Age-standardized rate per 100,000 (95% CI)
Both sexes	2015	13,453	55.1 (54.1–56.0)	55.4 (54.4–56.3)
	2016	14,018	56.6 (55.7–57.5)	57.4 (56.4–58.3)
	2017	14,058	56.0 (55.1–56.9)	57.0 (56.0–57.9)
	2018	13,593	53.3 (52.4–54.2)	54.2 (53.3–55.1)
	2019	13,256	51.1 (50.2–52.0)	52.0 (51.1–52.9)
Female	2015	8471	68.5 (67.0–69.9)	69.1 (67.6–70.5)
	2016	9115	72.7 (71.2–74.2)	74.1 (72.6–75.6)
	2017	9055	71.3 (69.8–72.7)	72.9 (71.4–74.4)
	2018	8653	67.0 (65.6–68.4)	68.6 (67.1–70.0)
	2019	8472	64.6 (63.2–65.9)	66.1 (64.7–67.5)
Male	2015	4982	41.3 (40.1–42.4)	41.3 (40.2–42.5)
	2016	4903	40.1 (39.0–41.2)	40.2 (39.1–41.3)
	2017	5003	40.4 (39.3–41.5)	40.7 (39.6–41.9)
	2018	4940	39.2 (38.1–40.3)	39.4 (38.3–40.5)
	2019	4784	37.3 (36.3–38.4)	37.7 (36.6–38.8)

*CI *Confidence interval

**Fig. 1:**
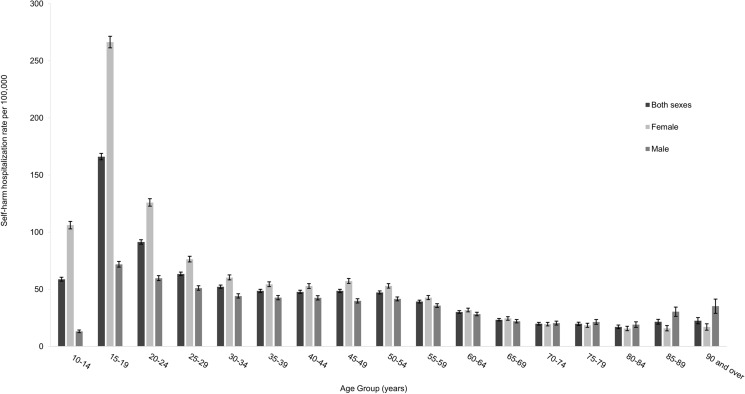
Self-harm hospitalization rate per 100,000 population, by sex and age group, Canada (excluding Quebec, Northwest Territories, and Yukon), 2015–2019. *Notes* Additional details in Supplemental Table 1

Five-year self-harm hospitalization rates by sex and age group are reported in Fig. 1. For both males and females, self-harm hospitalization rates peaked in the 15–19-year age group. Rates were significantly higher for females than males for age groups under 65 years old. In the two oldest age groups (85–89 and 90 years and older), males had higher rates than females.

### Self-harm hospitalization rate by rurality

**Fig. 2:**
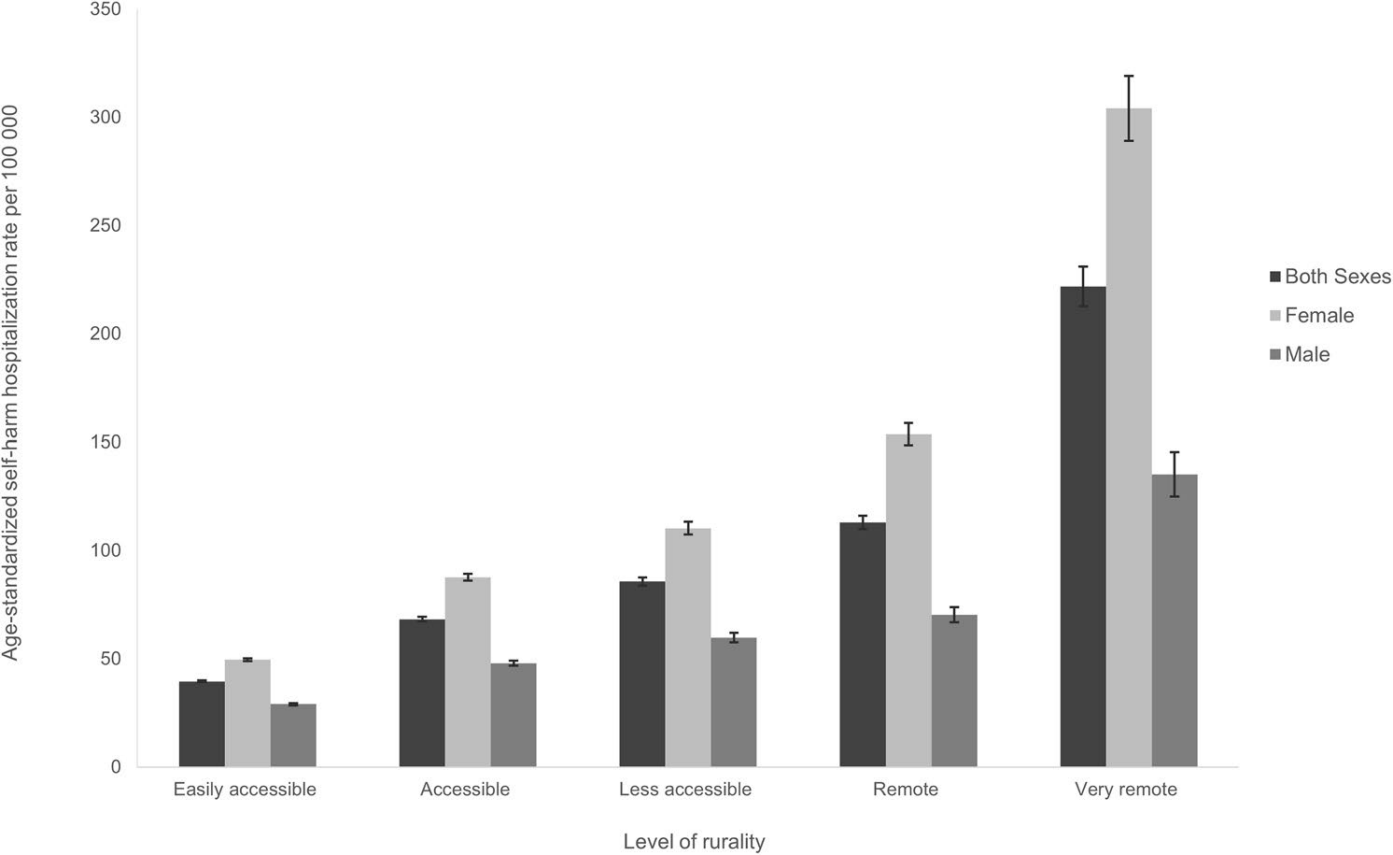
Age-standardized self-harm hospitalization rate per 100,000 population, by rurality, Canada (excluding Quebec, Northwest Territories, and Yukon), 2015–2019. *Notes* Additional details in Supplemental Table 2

We identified 66,241 self-harm hospitalizations between 2015 and 2019 that had census subdivision information, and so could be used for analyses by rurality. Age-standardized self-harm hospitalization rates were highest for males and females in very remote areas (Fig. 2). Across all levels of rurality, self-harm hospitalization rates were significantly higher for females than males. For each additional level of rurality, there was a significant increase in the rate of self-harm hospitalization for both sexes (Fig. 2).

Populations in very remote areas had a 6.5 times higher rate (an additional 215.9 self-harm hospitalizations per 100,000), compared with populations in easily accessible areas (Table 3). Compared with easily accessible areas, all other levels of rurality had higher rates of self-harm hospitalization for females, males, and both sexes combined (Table 3). Among females, the rate ratio was 7.5 times higher in very remote areas, compared with easily accessible areas; the rate among males in very remote areas was 5.0 times higher.

**Table 3:** Rate differences and rate ratio of self-harm hospitalizations for males and females, Canada (excluding Quebec, Northwest Territories, and Yukon), 2015–2019

	Level of rurality	Crude rate per 100,000 (95% CI)	Rate difference per 100,000 (95% CI)	Rate ratio (95% CI)
Both sexes	Easily accessible	39.5 (39.0–39.9)	Ref	Ref
	Accessible	65.8 (64.8–66.7)	26.3 (25.3–27.4)*	1.7 (1.6–1.7)^†^
	Less accessible	78.3 (76.7–80.0)	38.9 (37.1–40.6)*	2.0 (1.9–2.0)^†^
	Remote	102.8 (100.0–105.5)	63.3 (60.5–66.1)*	2.6 (2.5–2.7)^†^
	Very remote	255.4 (245.1–265.8)	215.9 (205.6–226.3)*	6.5 (6.2–6.8)^†^
Female	Easily accessible	49.3 (48.7–50.0)	Ref	Ref
	Accessible	84.2 (82.6–85.7)	34.8 (33.2–36.5)*	1.7 (1.7–1.8)^†^
	Less accessible	100.6 (97.9–103.3)	51.2 (48.5–54.0)*	2.0 (2.0–2.1)^†^
	Remote	141.8 (137.1–146.4)	92.4 (87.7–97.1)*	2.9 (2.8–3.0)^†^
	Very remote	369.2 (351.3–387.0)	319.8 (302.0–337.7)*	7.5 (7.1–7.9)^†^
Male	Easily accessible	29.3 (28.7–29.8)	Ref	Ref
	Accessible	47.1 (45.9–48.2)	17.8 (16.5–19.1)*	1.6 (1.6–1.7)^†^
	Less accessible	56.0 (53.9–58.0)	26.7 (24.6–28.8)*	1.9 (1.8–2.0)^†^
	Remote	64.7 (61.6–67.8)	35.4 (32.2–38.5)*	2.2 (2.1–2.3)^†^
	Very remote	147.1 (136.1–158.1)	117.9 (106.9–128.9)*	5.0 (4.7–5.4)^†^

*CI* Confidence interval

*Rate difference *p*-value < .0001, ^†^rate ratio *p*-value < .0001

**Fig. 3:**
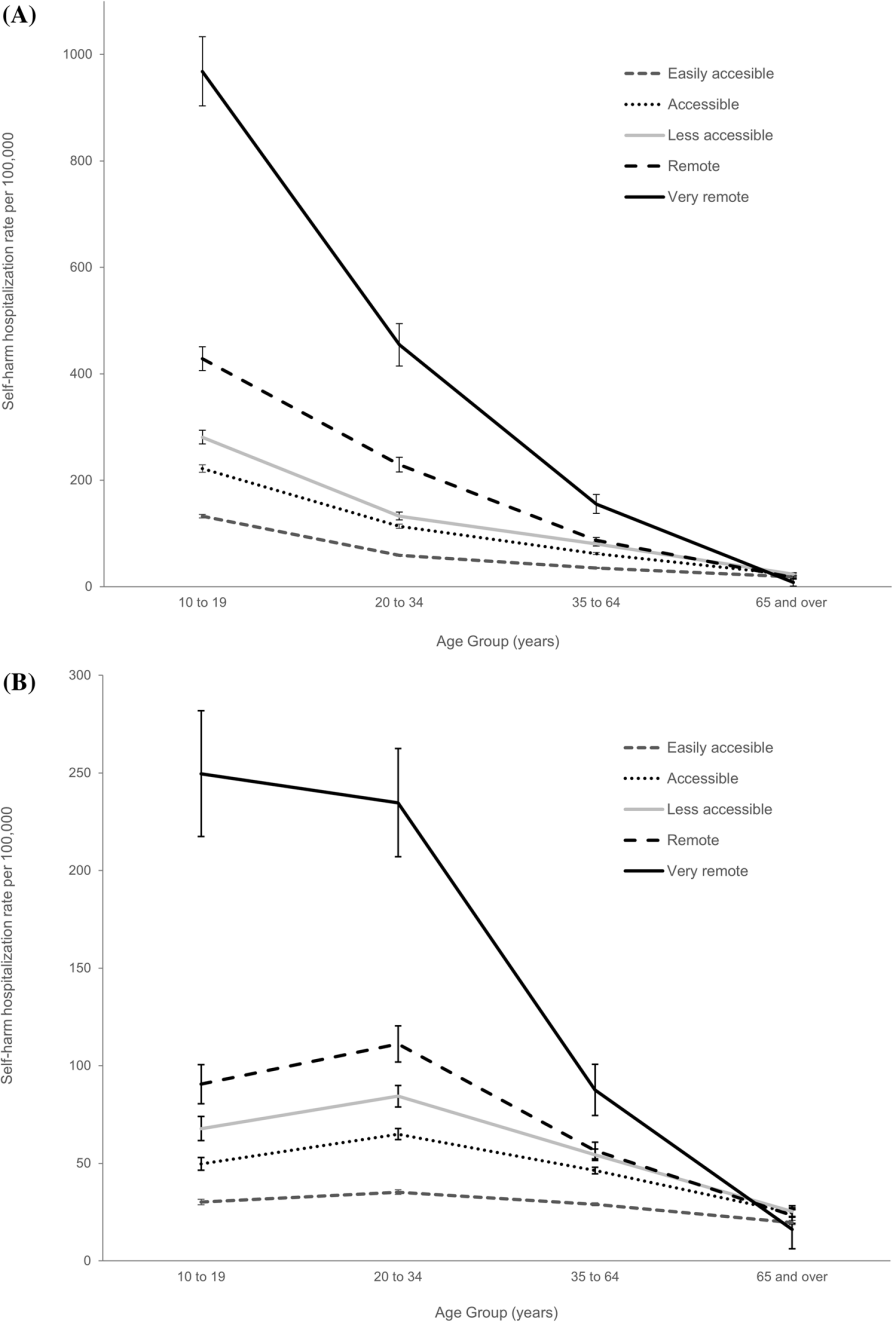
Self-harm hospitalization rate per 100,000 population for females (**A**) and males (**B**), by age group and level of rurality, Canada (excluding Quebec, Northwest Territories, and Yukon), 2015–2019. *Notes* Additional details in Supplemental Table 3

Figure 3 shows self-harm hospitalization rates for females and males, by age group and rurality. Rates increased with each level of rurality for all age groups except 65 or older. For both sexes, the largest rate differences were between very remote and easily accessible areas among 10–19 year-olds, followed by 20–34-year-olds (Fig. 3). For females, rates decreased with age across all levels of rurality. A similar pattern was found for males, except in the 10–19-year age group, who had similar or lower rates compared to males aged 20–35 years.

## Discussion

This study examined self-harm hospitalization rates by year, sex, age group, and rurality in Canada from 2015 to 2019. Age-standardized rates were relatively stable over the study period, with females having significantly higher rates than males each year. For both sexes, rates were highest among 15- to 19-year-olds. The age-standardized rate increased along a gradient of rurality, with the highest rates in the most remote areas. Although rurality affected both males and females, rate ratios were higher for females than males in more remote areas, compared with easily accessible areas. Differences were most pronounced for the 10–19 and 20–34 age groups.

These results reveal substantial disparities in self-harm between rural and urban areas in Canada, which aligns with evidence from British Columbia [16] and Ontario [17, 21]. The rural–urban gradient in self-harm is also similar to the national pattern of suicide mortality [12, 13]. The magnitude of the rate disparities in our study may have been exacerbated by the lack of adjustment for social and material deprivation, which is strongly associated with self-harm and suicide [24–26, 38, 39].

Although our results were consistent with several Canadian studies [16, 17, 21], research from Ireland and the United Kingdom revealed an inverse association between self-harm and rurality [24, 40]. That is, after controlling for deprivation, the risk of self-harm was higher in urban compared with rural areas. However, deprivation does not fully explain the spatial patterning in urban areas [41]. The contrasting trends in self-harm across the rural–urban continuum in high-income, English-speaking counties in Europe compared with Canada and the United States [42] suggests the influence of other factors that may differ between these contexts, such as firearm ownership and population density.

Across all levels of rurality, our results showed that self-harm hospitalization rates were higher for females, especially in rural and remote areas. This finding suggests that females in these communities may experience elevated risks compared to males and urban females, possibly due to factors such as chronic disease multimorbidity [43, 44] and experiences of violence. For example, women in rural and remote communities in Canada experience higher rates of police-reported intimate partner violence (IPV) [45, 46]. Although self-reported data indicates that the prevalence of IPV is similar across levels of rurality [46], IPV among rural and remote women may be more severe and chronic [46, 47]. The association between such forms of gender-based violence and self-harm [48, 49] may play a role in the elevated rates of self-harm hospitalizations for females in rural and remote areas.

Although hospitalization rates were higher among females, males have higher rates of suicide mortality [10, 21, 50]. Lower self-harm hospitalization rates among males may be due, in part, to males using more lethal means (for instance, firearms) in suicide attempts. [51]. Examining self-harm case fatality may further disentangle possible sex-specific relationships between rurality and suicide means. Another possible explanation for the sex differences in hospitalization is that cultural norms about masculinity may dissuade males in rural communities from seeking care for self-harm [52]. Even so, the risk of self-harm hospitalization was higher for rural than urban males, which may be related to differences in access to services between settings [53, 54]. Rural males may be more receptive to interventions which are short-term, collaborative, and oriented toward problem-solving [55].

In our study, rural adolescents (aged 10–19) had the highest rates of self-harm hospitalization and the largest rate differences and ratios. Adolescents experience a constellation of risk factors for self-harm including depressed mood, impulsivity, conflict with peers and family, childhood maltreatment, and psychological distress [56–58]. Our results align with other North American studies that have found self-harm hospitalization rates to be higher among rural than urban youth [16, 17, 59]. Rural adolescents may be a particularly vulnerable, owing to limited access to mental health services [60], stigma associated with seeking help, and lack of awareness of mental health resources [61].

Interventions that may reduce self-harm among rural youth include increasing access to mental health services [62], public health campaigns that address norms related to help-seeking [63], and efforts to encourage participation extracurricular activities to foster bonds with peers [64]. As primary care and emergency departments are often the first point of contact for adolescents who self-harm [65], clinical and system interventions for self-harm such as safety planning and increasing access to care, should be tailored to the differential risks across geographic contexts.

### Strengths and limitations

A strength of this study is that we used a definition of self-harm based on an internationally standardized classification system and a multifaceted, scaled definition of rurality. We chose the Index of Remoteness because it is based on population size and accessibility to other regions. Thus, we were able to distinguish between rural and remote regions, unlike studies of self-harm and suicide using binary measures of rurality [14, 17, 19, 66], which can obscure differences between diverse rural contexts. This is an important consideration in Canada, one of the least densely populated countries in the world, where residents of remote communities may experience place-specific risks. Another strength of our study is that the dataset had comprehensive coverage for most provinces and territories.

Several limitations should be considered when interpreting the results. Not all cases of self-harm hospitalization were included in our analyses. We did not use data from Quebec, Northwest Territories, or Yukon. These three jurisdictions have proportionately large Indigenous populations in remote regions [67], and some rural and northern Indigenous communities have high rates of self-harm compared with the general population [68]. Consequently, our estimate of the national self-harm hospitalization rate for the most remote areas may be less representative than our estimate of the national rate for urban areas.

The DAD does not include patients who visited the emergency department because of self-harm but were not hospitalized. Therefore, we likely captured the more medically serious non-fatal incidents, but not all episodes of hospital-treated self-harm. In the suicide surveillance indicator framework used in Canada, self-harm hospitalizations and suicide deaths are recognized as less frequent, but more harmful, outcomes [5, 68]. Other datasets based on emergency department visits [17] and population surveys [69] provide evidence about more prevalent but less severe outcomes such as suicidal ideation [70]. This “iceberg” model [68, 71] requires indicators across a continuum of lethality in order to describe the scope and scale of suicidality in the population. Our study provides evidence on only one outcome in this model.

Owing to the nature of the data, we were unable to analyze co-variates such as income, marginalization, or deprivation, either at the individual or area level. This meant that we could not adjust for the confounding effects of these social determinants, which are associated with self-harm, especially in high-income countries [38, 39]. Examining relationships between self-harm and chronic diseases or multimorbidity of physical and mental illnesses was also not within the scope of our study. These factors are known to be associated with suicidal behaviour [44], increase the odds of seeking hospital care [72], and may be more prevalent among females and rural populations [43]. Even without adjusting for these confounders, our results were consistent with recent evidence from Canada’s largest province (Ontario) which indicated that, after controlling for income, immigration status, marginalization, and chronic conditions, males and females in rural areas were significantly more likely to attempt suicide than were their urban counterparts, [21]. Future research with national data could clarify relationships between rurality, socio-economic deprivation, chronic disease status, and sex differences in self-harm.

Another limitation was the difficulty of ascertaining injury intent, which may have resulted in misclassifications of those who presented to the hospital [73]. This may have led to an underestimation of self-harm in the population, particularly for self-harm by poisoning, where the intent of an overdose may be difficult to determine [74]. To minimize the effects of the pandemic on our results, DAD data collected after March 31, 2020 were not included in this study. Additional analyses to understand the impact of the pandemic on self-harm in Canada is needed. Early evidence on self-harm-related emergency department visits suggests that the pandemic may have had a differential impact on urban and rural populations [70].

## Conclusion

This population-based study produced subnational estimates of self-harm hospitalization by rurality in Canada. A rural–urban gradient in self-harm hospitalization was evident—rates were lowest in urban areas and highest in the most remote communities. Rates were highest among females aged 15–19 and females in very remote areas. These findings underscore the need to design population-based and targeted interventions for rural and remote contexts. Ongoing public health surveillance is required to understand the impact of the pandemic on self-harm, and the contribution of different methods of self-harm to risk of hospitalization and mortality.

### Supplementary Information

Below is the link to the electronic supplementary material.

Supplementary file1 (PDF 277 kb)

## Data Availability

The data used in this study was made available through an agreement between the Public Health Agency of Canada and the Canadian Institute for Health Information.
