# Supplementary material for: Self-harm and rurality in Canada: an analysis of hospitalization data from 2015 to 2019
Source: Soc Psychiatry Psychiatr Epidemiol. 2023 Apr 8;58(8):1161–70. doi: 10.1007/s00127-023-02463-7 (PMC10081931; doi:10.1007/s00127-023-02463-7)
Supplement: Supplementary file 1 — Supplementary file1 (PDF 277 kb) [file 127_2023_2463_MOESM1_ESM.pdf]

Supplementary Tables

Title
Self-harm and rurality in Canada: an analysis of hospitalization data from 2015-2019

Authors
Newsha Mahinpey MPH 1, 2, Nathaniel J. Pollock PhD 1, 3, Li Liu PhD 1, Gisèle Contreras MSc 1, Wendy Thompson MSc 1

Supplemental Table 1 Age-specific self-harm hospitalization per 100,000 population by sex and age group, Canada (excluding Quebec, Northwest Territories, and Yukon) 2015-2019

	**Both sexes combined**	**Female**	**Male**
**Age Group**	(95%CI)	(95%CI)	(95%CI)
**10-14**	58.8 (57.1-60.5)	106.2 (102.9-109.4)	13.2 (12.1-14.3)
**15-19**	166.2 (163.4-168.9)	266.4 (261.3-271.4)	71.9 (69.3-74.4)
**20-24**	91.4 (89.5-93.3)	126.0 (122.7-129.2)	59.7 (57.6-61.9)
**25-29**	63.5 (61.9-65.0)	76.4 (73.9-78.8)	51.2 (49.2-53.1)
**30-34**	52.2 (50.8-53.7)	60.4 (58.2-62.5)	44.2 (42.3-46.0)
**35-39**	48.6 (47.2-50.0)	54.4 (52.3-56.4)	42.7 (40.8-44.5)
**40-44**	47.8 (46.3-49.2)	52.8 (50.7-54.9)	42.5 (40.6-44.4)
**45-49**	48.6 (47.2-50.0)	57.3 (55.2-59.4)	39.8 (38.0-41.6)
**50-54**	47.3 (46.0-48.6)	53.0 (51.0-55.0)	41.6 (39.8-43.3)
**55-59**	39.2 (38.0-40.5)	42.8 (41.0-44.5)	35.7 (34.0-37.3)
**60-64**	30.1 (29.0-31.2)	31.8 (30.2-33.5)	28.3 (26.7-29.9)
**65-69**	23.3 (22.2-24.4)	24.5 (23.0-26.1)	22.0 (20.5-23.6)
**70-74**	19.9 (18.8-21.1)	19.5 (18.0-21.1)	20.4 (18.7-22.1)
**75-79**	19.9 (18.5-21.2)	18.5 (16.7-20.3)	21.4 (19.3-23.5)
**80-84**	17.1 (15.6-18.6)	15.6 (13.7-17.6)	19.0 (16.6-21.5)
**85-89**	21.6 (19.5-23.7)	15.9 (13.5-18.2)	30.4 (26.4-34.4)
**90 and over**	22.5 (19.8-25.3)	17.0 (14.1-19.8)	35.2 (29.0-41.5)

Abbreviations: CI= Confidence interval

Supplemental Table 2 Age-standardized self-harm hospitalization rate per 100,000 population by level of rurality, Canada (excluding Quebec, Northwest Territories, and Yukon) 2015-2019

**Level of Rurality**	**Both sexes combined (95%CI)**	**Female (95%CI)**	**Male (95%CI)**
**Easily accessible**	39.6 (39.2-40.0)	49.6 (48.9-50.3)	29.1 (28.6-29.6)
**Accessible**	68.4 (67.4-69.4)	87.68 (86.1-89.3)	48.1 (46.9-49.3)
**Less accessible**	85.7 (83.9-87.6)	110.4 (107.4-113.4)	59.8 (57.6-62.0)
**Remote**	113.1 (110.0-116.2)	153.7 (148.6-158.8)	70.4 (67.0-73.8)
**Very Remote**	221.8 (212.6-231.0)	304.2 (289.2-319.2)	135.2 (125.0-145.5)

Abbreviations: CI= Confidence interval

Supplemental Table 3 Age specific self-harm hospitalization rate per 100,000 population for females and males by age group and level of rurality, Canada (excluding Quebec, Northwest Territories, and Yukon) 2015-2019

Age Group	Level of Rurality	Female rate (95%CI)	Female RR (95%CI)	Male rate (95%CI)	Male RR (95%CI)
**10-19**	Easily accessible	132.4 (129.2-135.6)	ref	30.2 (28.7-31.7)	ref
	Accessible	221.8 (214.7-228.9)	1.7 (1.6-1.7)	49.7 (46.5-53.0)	1.6 (1.5-1.8)
	Less accessible	280.1 (268.0-294.0)	2.1 (2.0-2.2)	67.8 (61.6-74.0)	2.2 (2-2.5)
	Remote	428.2 (405.8-450.7)	3.2 (3.1-3.4)	90.6 (80.5-100.6)	3.0 (2.7-3.4)
	Very Remote	968.3 (903.2-1033.3)	7.3 (6.8-7.9)	249.7 (217.5-281.8)	8.3 (7.2-9.5)
**20-34**	Easily accessible	58.7 (57.2-60.2)	ref	35.2 (34.1-36.4)	ref
	Accessible	113.5 (109.7-117.3)	1.9 (1.9-2.0)	65.0 (62.2-67.8)	1.8 (1.7-2.0)
	Less accessible	132.7 (125.5-139.9)	2.3 (2.1-2.4)	84.4 (78.9-89.9)	2.4 (2.2-2.6)
	Remote	229.0 (215.2-242.8)	3.9 (3.7-4.2)	111.2(101.9-120.5)	3.2 (2.9-3.5)
	Very Remote	454.4 (414.8-494.0)	7.7 (7.1-8.5)	234.8 (207.0-262.5)	6.7 (5.9-7.5)
**34-64**	Easily accessible	34.9 (34.1-35.8)	ref	29.0 (28.2-29.8)	ref
	Accessible	62.1 (60.2-64.1)	1.8 (1.7-1.9)	46.3 (44.6-48.0)	1.6 (1.5-1.7)
	Less accessible	80.2 (76.6-83.7)	2.3 (2.2-2.4)	54.4 (51.5-57.3)	1.9 (1.8-2.0)
	Remote	87.2 (81.9-92.5)	2.5 (2.3-2.7)	56.6 (52.3-60.8)	2.0 (1.8-2.1)
	Very Remote	155.3 (137.4-173.1)	4.4 (4.0-5.0)	87.6 (74.5-100.7)	3.0 (2.6-3.5)
**65+**	Easily accessible	18.3 (17.4-19.3)	ref	19.7 (18.6-20.8)	ref
	Accessible	22.3 (20.6-24.0)	1.2 (1.1-1.3)	24.7 (22.8-26.6)	1.3 (1.1-1.4)
	Less accessible	23.4 (20.7-26.1)	1.3 (1.1-1.4)	25.4 (22.4-28.3)	1.3 (1.1-1.5)
	Remote	17.1 (13.7-20.5)	0.9 (0.8-1.1)	23.3 (19.2-27.4)	1.2 (1.0-1.4)
	Very Remote	8.4 (1.0-15.7)	0.5 (0.2-1.1)	16.2 (6.2-26.3)	0.8 (0.4-1.5)

Abbreviations: RR: Rate ratio; CI: confidence interval
